# Magnitude of *Helicobacter pylori* and associated risk factors among symptomatic patients attending at Jasmin internal medicine and pediatrics specialized private clinic in Addis Ababa city, Ethiopia

**DOI:** 10.1186/s12879-019-3753-5

**Published:** 2019-02-06

**Authors:** Gemechu Shiferaw, Dessie Abera

**Affiliations:** 1 School of medicine, Addis Ababa University, Addis Ababa, Ethiopia; 2 Department of medical laboratory Sciences, Addis Ababa University, Addis Ababa, Ethiopia

**Keywords:** Peptic ulcer, Dyspepsia, Bacteria, Stool antigen, Ethiopia

## Abstract

**Background:**

More than 50% of the people are infected worldwide with *H. pylori* which causes significant public health morbidity and mortality. The distribution is quite different from country to country. Hence, early information is very important to prevent upper gastrointestinal complications. The current study aimed to assess the magnitude of *H. pylori* and associated risk factors among symptomatic patients attending at Jasmin internal medicine and pediatrics specialized private clinic from August 2017 until May 2018 in Addis Ababa city, Ethiopia.

**Methods:**

A cross-sectional study was conducted among 487 patients with upper gastrointestinal tract complaints attending at Jasmin internal medicine and pediatrics specialized private clinic from August 2017 until May 2018. Convenient sampling technique was used to enroll participants. Information regarding to risk factors was assessed using structured questionnaire. Stool samples were collected for *H. pylori* antigen test. Data was entered and analyzed using SPSS version20 statistical software and a *p*-value less than 0.05 was considered as statistically significant.

**Results:**

The overall prevalence of *H. pylori* among participants using stool antigen was 36.8% (*n* = 179/487). Regarding to family income status, those who have low monthly income were more likely to be infected with *H. pylori* infection (AOR = 6.056, CI 95% = 1.603–22.881, *P* = 0.037). In addition, families with low educational level were more likely to be infected with *H. pylori* infection than higher level education (AOR = 4.150, CI95% = 1.059–16.270, *P* = 0.041). Number of family members in the house-hold, type of toilet they used and source of drinking water were not significantly associated with *H. pylori* infection.

**Conclusions:**

The prevalence of *H. pylori* infection was 36.8% and it was related to low income and low education levels. This finding calls for improving the socioeconomic status of the community. Moreover, further studies are needed to investigate potential risk factors for *H. pylori* infection.

## Background

*Helicobacter pylori* (*H. pylori*) infection is globally accepted as a major public health problem, with high burden in African countries [1]. In most cases, it is the cause of acute and chronic gastritis and highly associated to gastric cancer and peptic ulcer [2]. The bacterium causes peptic ulcer by eroding the epithelial tissue of the stomach and the upper part of the small intestine, which makes the stomach acid to get through to the sensitive lining layer. The inflammation in the lining of the stomach or duodenum is the combined effect of stomach acid and *H. pylori*, which leads to peptic ulceration [3]. Although the route of transmission of *H. pylori* infection is not clearly known, some evidences indicate that fecal contaminated water and food, faecal-oral contact and kissing are source of infection [4].

*Helicobacter pylori* infection is apparent in resource limited countries than in economically developed countries and the distribution varies between different communities and geographical locations [5]. About 50% of the population is infected with *H. pylori* in affluent countries, whereas this percentage rises to 80% in developing countries [1], and the prevalence in Ethiopia is in the range of 48–95% [6].

The infection is often acquired during childhood and persists throughout life; remaining dormant for an extended period of time, and disease manifestations not appearing up to adulthood. The majorities of infected people are unaware of their infection status and remain to be infectious to others. Only some people develop illness, most of the time in adulthood [7]. Contributing factors for *H. pylori* infection are related to socio-demographic characteristics, personal and environmental hygiene, life style of the population, genetic predisposition and socioeconomic status [8].

The presence of *H*. pylori by itself is not a disease, but a condition, that causes the probability of developing various clinical disorders of the upper gastrointestinal tract. So, it should be integrated with the clinical history of an individual, which is very important to prevent the development of gastric cancer and peptic ulcer [9]. In addition, stool antigen testing is recommended for the diagnosis of *H. pylori* infection in patients with upper gastrointestinal symptoms [10]. In Ethiopia, many studies were used serology rapid tests but few data on stool antigen test which indicates active infection. Therefore, this study aimed to assess the prevalence of *H. pylori* and associated risk factors among symptomatic patients at Jasmin internal medicine and pediatric specialized private clinic, Addis Ababa, Ethiopia.

## Methods

### Study design, period and area

A cross-sectional study was conducted to determine the magnitude of *H. pylori* among symptomatic patients attending Jasmine internal medicine and pediatrics specialized clinic from August 2017 until May 2018. Jasmine internal medicine and pediatrics specialized clinic is a private clinic located in Addis Ababa city, Kolfe sub city. All the study participants were urban population. Patients with upper gastrointestinal tract complaints are frequently tested in the clinic, using stool antigen tests.

### Sample size determination and sampling technique

Single population proportion formula, n = (Zα/2)^2^ * P (1- P) /d^2^), was used to calculate the minimum sample size and the prevalence (p) taken was 48.7%, from previous study in Assosa, Ethiopia (10), with a marginal error of 5, and a 95% confidence interval. Based on this calculation the sample size was 383. Nevertheless, we have collected 487 samples. All patients who have symptoms of *H. pylori* infection and who were volunteers to participate were included**.** Patients treated with any antibiotics like, omeprazole, amoxicillin, clarithromycin, bismuth subsalicylate, and lansoprazole, within the last 4 weeks, were excluded.

### Data collection procedure

All age groups were included in the study, before data collection; written informed consent was obtained from study participants and children’s parents, for those who were below 18 years old. The purpose of the study, the study procedures, possible risks/benefits, the rights and responsibilities of participants including their right to withdraw from the study at any time, was described.

Interviewer lead questionnaire was filled by parents/guardians. Once all questions had been answered satisfactorily, parents/guardians who were interested in enrolling their children and themselves in the study were asked to sign an informed consent.

The parents were asked to complete a short questionnaire regarding to their daily habits, household information, and potential sources of infection. After this, the children and the parents were provided with a clean, dry, disinfectant free, wide mouthed plastic container to collect about 10 g of stool specimen into the container for *H. pylori* antigen test. Instruction was given how to prevent contamination of the stool with water and urine.

### Laboratory analysis

#### Stool antigen test

Stool samples requested from each participating patient were collected in leak-proof containers. A small portion of the stool sample was transferred to a vial with diluents, vigorously agitated for 15 s and after that two to three drops were added into the round window of the test cassette. The results were interpreted after 15 min (according to the manufacturer’s instruction) and we interpreted the results based on the appearance of colored lines across the central window of the cassette. Appearance of two lines, C (control) and T (test), indicates a positive test, and appearance of only one line, C (control) indicates a negative result.

### Data analysis and interpretation

The completed data collection tool was checked for completeness and consistency and, was coded by the principal investigators. Data cleanup was performed to check for accuracy and consistencies. Any identified error was corrected immediately. Statistical analysis was performed using SPSS version 20. Descriptive statistics was used to describe the socio-demographic data, binary and multiple regression tests (odds ratio/adjusted odds ratio) were employed to assess the significant association between *H. pylori* and risk factors. A *P*-value of less than 0.05 was considered as statistically significant.

## Results

### Socio-demographic characteristics of study participants

A total of 487 patients participated in this study. The mean age of participants was 26.8 ± 16.1SD (age range 3–75 years old). Majority of the study participants were females, 57.3% (279/487). All the study participants were urban dwellers. About 85.6% of the participants’ had a monthly between 3000 and 3500 Ethiopian birr. Regarding to educational status, university educational level accounted for the highest percentage, 90.8% (442/487), as demonstrated below (Table 1).

**Table 1:** Socio-demographic characteristics of symptomatic patients at Jasmin internal medicine and pediatrics specialized clinic, Addis Ababa, Ethiopia, 2018 (*n* = 487)

Variables		Frequency	Percent (%)
Sex	Female	279	57.3
	Male	208	42.7
Age category in years	3–10	93	19.1
	11–18	63	12.9
	19–26	112	23.0
	27–34	79	16.2
	35–42	65	13.3
	43–50	30	6.2
	> 50	45	9.2
Income	1000–1500	5	1.0
	2000–2500	38	7.8
	3000–3500	417	85.6
	> 4000	27	5.5
Family number	1	26	5.3
	2	66	13.6
	3	252	51.7
	4	118	24.2
	5	22	4.5
	6	2	4
Educational status	Primary	13	2.6
	High school	32	6.6
	University	442	90.8

*COR* Crude odds ratio, *CI* Confidence interval

### Distribution of *H. pylori* infection and its association with risk factors

The prevalence of *H. pylori* in this study was 36.8% (179/487). The proportion of *H. pylori* was higher in females than males (38.7% versus 34.1%) but the difference was not statistically significant (*p* = 0.301). Regarding family monthly income status, those who earn 1000–1500 and 2000–2500 Ethiopian birr were more likely to be affected with *H. pylori* infection (COR = 7.150, CI 95% = 1.927–26.524, *p* = 0.003 and COR = 9.350,CI 95% = 2.589–33.769, *p* = 0.001) compared to those who earn3000–3500 Ethiopian birr (*p* > 0.05). Concerning educational status, primary level and secondary level education of the participants was significantly associated with *H. pylori* infection compared to higher level education (COR = 6.555, CI95% = 1.777–24.1777, *p* = 0.005 and COR = 3.277, CI95% = 1.560–6.885, *p* = 0.002) respectively.

The highest percentage, 46.7% (14/30), of *H. pylori* infections was noticed in the age group of 43–50 years old participants, followed by in the age group of 27–34 years old (40.5%). Prevalence of *H. pylori* infection was slightly higher, 39.8% (41/103) among participants who did not wash their hands than among participants who washed their hands after using toilet, 35.9% (138/384). Number of family members, type of toilet they used and source of drinking water were not associated with *H. pylori* infection (*p* > 0.05) as showed (Table 2).

**Table 2:** Distribution of *H. pylori* infection and its association with risk factors among symptomatic patients at Jasmin internal medicine and pediatrics specialized clinic, Addis Ababa, Ethiopia, 2018(n = 487)

Variables		Total	Stool antigen				
			Positive	Negative	COR	CI 95%	*P*-value
Sex	Female	279	108(38.7)	171(61.3)	1.219	0.838–1.772	0.301
	Male	208	71(34.1)	137(65.9)	1		
Age category	3–10	93	30(32.3)	63(67.7)	0.863	0.408–1.826	0.700
	11–18	63	24(38.1)	39(61.9)	1.115	0.504–2.469	0.788
	19–26	112	41(36.6)	71(63.4)	1.047	0.509–2.153	0.901
	27–34	79	32(40.5)	47(59.5)	1.234	0.578–2.633	0.587
	35–42	65	22(33.8)	43(66.2)	0.927	0.418–2.059	0.853
	43–50	30	14(46.7)	16(53.3)	1.586	0.618–4.067	0.337
	> 50	45	16(35.6)	29(64.4)	1		
Income	1000–1500	21	13(61.9)	8(38.1)	7.150	1.927–26.524	0.003
	2000–2500	25	17(68.0)	8(32.0)	9.350	2.589–33.769	0.001
	3000–3500	414	144(34.8)	270(65.2)	2.347	0.870–6.327	0.092
	> 4000	27	5(18.5)	22(81.5)	1		
Family number	1–3	345	127(36.8)	218(63.2)	1		
	4–6	142	52(36.6)	90(63.4)	0.992	0.661–1.488	0.968
Education status	Primary	13	10(76.9)	3(23.1)	6.555	1.777–24.177	0.005
	High school	32	20(62.5)	12(37.5)	3.277	1.560–6.885	0.002
	University	442	149(33.7)	293(66.3)	1		
Type of toilet	Flush	413	154(37.3)	259(62.7)	1		
	Pit latrine	74	25(33.8)	49(66.2)	0.858	0.509–1.445	0.565
Water source	Tap water	429	163(38.0)	266(62.0)	1.532	0.660–3.558	0.321
	Boiled water	30	8(26.7)	22(73.3)	0.909	0.287–2.877	0.871
	Bottle water	28	8(28.6)	20(71.4)	1		
Hand wash after toilet	Yes	384	138(35.9)	246(64.1)	0.488	0.543–1.325	0.470
	No	103	41(39.8)	62(60.2)	1		

Multivariate analysis was performed for these factors that showed significant association at the crude odds ratio calculation. The association remains significant between *H. pylori* infection, income status and educational status (Table 3).

**Table 3:** Adjusted odds ratio for monthly income and education with *H. pylori* infection among symptomatic patients attending at Jasmin internal medicine and pediatrics specialized clinic, Addis Ababa, Ethiopia, 2018 (*n* = 487)

Variables		Stool antigen					
		Total	Positive n (%)	Negative n (%)	AOR	CI 95%	*P*-value
Income level	1000-1500 EB	21	13(61.9)	8(38.1)	4.316	1.093–17.038	0.037
	2000-2500 EB	25	17(68.0)	8(32.0)	6.056	1.603–22.881	0.008
	3000–3500 EB	414	144(34.8)	270(65.2)	2.222	0.823–5.997	0.115
	> 4000 EB	27	5(18.5)	22(81.5)	1		
Education level	Primary	13	10(76.9)	3(23.1)	4.150	1.059–16.270	0.041
	High school	32	20(62.5)	12(37.5)	2.280	1.025–5.067	0.043
	University	442	149(33.7)	293(66.3)	1		

*AOR* Adjusted odds ratio, *CI* Confidence interval, *EB* Ethiopian birr (1USD = 27.823 EB)

## Discussion

The prevalence of *H. pylori* varies across the globe and this is partly due to socioeconomic difference [4]. *H. pylori* infection in symptomatic patients is highly associated with an increased risk of developing gastric cancer [11]. In this study, the overall prevalence of *H. pylori* was 36.8% (179/487) which was lower than studies done in Ethiopia, Gondar (85.6%), Hawassa (62.3%) and Asosa (48.7%) [12–14] respectively, while it was higher than reported by Seid et al (30.4) [15]. The reason for this variation could be due to difference in sample size, laboratory testing methodology, good sanitation practice at individual and community level. Moreover, the above studies were used antibody test which may lead to over estimation of prevalence.

Our finding also less than studies reported in Brazil (78.8%) [16], Beninese (74%) [17], and China (48%) [18], whereas it was higher than studies conducted in Switzerland (18.9%), Denmark (22.1%), New Zealand (24.0%), Australia (24.6%), and Sweden (26.2%) [1]. The difference might be due to variation in study design and large sample size in their study and the fact that *H. pylori* infection has strong association with socioeconomic status. In other words, in countries with high socioeconomic status, there is good hygiene practice, environmental sanitation and adequate provision of safe water which can reduce the prevalence rate.

There is no consensus on the state of being male or female as a risk factor for *H. pylori* infection but a study suggested that physiological difference between female and male may affect the mechanism of immune response to the pathogenesis of the bacterium [19]. In the current study, the proportion of *H. pylori* was higher in females, 38.7% (108/279) than males, 34.1% (71/208), but the difference was not statistically significant (*P* > 0.05). The number of female participants was greater than male participants; this might be the reason why the prevalence of *H. pylori* in female participants was higher than males. However, further large scale studies on sex specific *H. pylori* infection is needed to explain this variation. This finding was consistent with previous studies done in Ethiopia [12, 13, 15, 20] and Kenya [21]*.* In contrast, other studies showed that *H. pylori* infection was significantly higher in males than females [19, 22].

Mostly people get their primary *H. pylori* infection during childhood and stay infected during their life time, however it is not clear when the acquisition is takes place [23, 24]. Our study indicated high proportion of *H. pylori* infection in the age groups of 43–50 years old 46.7% (14/30), however, this difference was not significant (*p* > 0.05). This was comparable to, previous studies conducted in Ethiopia, showed no significant association between age and *H. pylori* infection [13, 25]. Contrary to this, significant association between age and *H. pylori* infection was reported in Ethiopia, Butajira [26]. Source of drinking water has been noticed as one of the contributing factors for the high prevalence of *H. pylori* infection, this is apparent in developing countries where there is lack of access to clean water and poor sewerage system [27]. The relationship between source of drinking water and *H. pylori* infection was not statistically significant (*p* > 0.05). This result was similar to previous report in Ethiopia [13] and China [28].

Education and income status is strongly associated with *H. pylori* infection and this is one of the reasons to the wide variation in the prevalence of this infection between countries [29]. Countries with low socioeconomic status, like Ethiopia, there is poor personal hygienic practice, poor waste disposal system, crowded living conditions and lack of clean and safe water which are known to make individuals susceptible for infection [30] . In our finding, *H. pylori* infection was significantly higher in low–income households (*p* = 0.008), which was similar to the above concept and this was in agreement with other reports that have identified low income as a risk-factor predisposing to infection [31]. However, this was incongruent to a study done in Benine [17]. The current study indicated that low educational level was showed statistical significant association with prevalence of *H. pylori* infection (*p* = 0.041). This association could be due to the fact that inadequate education or low education level has a significant impact on personal hygiene and environmental hygiene and play a great role in the rise of the prevalence of *H. pylori* infection [21, 25, 27]. In contrast, previous study reported from Ethiopia, Assosa revealed that education level was not associated with *H. pylori* infection [13].

### Limitation

The limitation of this study was unable to include asymptomatic individuals, which confines the true prevalence of *H. pylori* infection.

## Conclusion

In conclusion, our study indicated that low income level and low educational status were significantly associated with *H. pylori* infection, but it was not related with source of drinking water, age and gender. Improving the socioeconomic status of the community will reduce the transmission and the burden of *H. pylori* infection. Further studies are needed to investigate potential risk factors for *H. pylori* infection.

## Abbreviations

AOR: Adjusted odds ratio
COR: Crude odds ratio
